# Extraperitoneal Robot-Assisted Radical Prostatectomy Using Hinotori™ Surgical Robot System: Report of First Series of Seven Cases

**DOI:** 10.7759/cureus.92173

**Published:** 2025-09-12

**Authors:** Jun Teishima, Yukari Bando, Akihisa Yao, Koki Maeda, Katsuya Sato, Kotaro Suzuki, Takuto Hara, Tomoaki Terakawa, Koji Chiba, Hideaki Miyake

**Affiliations:** 1 Division of Urology, Department of Surgery Related, Kobe University Graduate School of Medicine, Kobe, JPN

**Keywords:** extraperitoneal approach, hinotori™ surgical robot system, prostate cancer, robot-assisted radical prostatectomy, robot-assisted surgery

## Abstract

Objectives: The Hinotori™ surgical robot system (HSRS) (Medicaroid, Kobe, Japan), a newly launched domestic platform in Japan, has already been utilized in many robot-assisted surgeries. This study aimed to assess the perioperative outcomes of the first series of seven cases that underwent robot-assisted radical prostatectomy (RARP) with an extraperitoneal approach (EP) using HSRS.

Methods: A total of seven consecutive patients with localized prostate cancer who underwent RARP with EP using the HSRS between January and May 2025 at our institution were included in this study. Their comprehensive perioperative outcomes were retrospectively analyzed.

Results: The median age and the body mass index (BMI) of the patients were 69 years and 21.9 kg/m², respectively. RARP with EP could be completely performed on all patients without conversion to either a transperitoneal approach or open surgery. The median operative time, the time using the robotic system, the estimated blood loss, and the length of the indwelling urethral catheter were 180 min, 96 min, 100 mL, and seven days, respectively. No patient experienced major perioperative complications requiring invasive treatment or blood transfusion.

Conclusions: Despite being a small case series, this is the first report of the perioperative finding that RARP with EP using the HSRS is expected to provide equivalent perioperative outcomes compared to RARP with transperitoneal (TP).

## Introduction

The introduction of robot-assisted surgery into routine clinical practice has led to marked changes in minimally invasive surgery. While the da Vinci™ (Intuitive, Sunnyvale, California) surgical system (DVSS) has played the central role in robot-assisted surgery in Japan, following this, the hinotori™ surgical robot system (HSRS) was developed in 2019 by the Medicaroid Corporation (Kobe, Japan) as the first domestic robot-assisted surgical system, and it was cofunded by the Sysmex Corporation and Kawasaki Heavy Industries (Kobe, Japan) [1]. The HSRS has already been utilized in many robot-assisted surgical procedures for urological surgeries, including partial nephrectomy [2], radical nephrectomy [3], adrenalectomy [4], radical nephroureterectomy [5], radical cystectomy [6], and pyeloplasty [7]. The first robot-assisted surgery using the HSRS was robot-assisted radical prostatectomy (RARP), and the perioperative outcomes of its initial cases were reported [8]. Many urologic surgeons usually perform RARP using the transperitoneal approach (TP) because it allows enough distance between trocars to provide adequate working space [9]. In addition, RARP using the extraperitoneal approach (EP) has been demonstrated. EP has an advantage in that it can be performed without concern for intra-abdominal adhesions, even in patients with a history of intra-abdominal surgery [10], and it can contribute to decreasing intra-abdominal complications [9,11]. Unlike TP, EP does not require a steep Trendelenburg position, making it effective for patients with underlying diseases such as severe glaucoma or cerebral aneurysms that would contraindicate this position [12,13]. However, EP has a smaller working space than TP, and its disadvantage is considered to be the tendency for the arms of the robot to interfere with each other. Thus, there have been many reports on the characteristics of RARP with EP using the DVSS, but there have been no reports on RARP with EP using the HSRS. In this report, we describe our initial experience of RARP with EP using the HSRS.

## Materials and methods

This study included a total of seven consecutive patients with localized prostate cancer who received RARP with EP between January and May 2025 at our institution. This study was conducted in accordance with the Declaration of Helsinki (as revised in 2013). The concept and design of this study were approved by the Research Ethics Committee of Kobe University (approval number B240145), and the need to obtain written informed consent for involvement in the present study from all of the included patients was waived due to its retrospective design.

All data on the clinicopathological and perioperative findings of the patients were obtained from their medical records at our institute. The indication for RARP was clinically localized prostate cancer free from metastasis. Extended pelvic lymph node dissection (ePLND) was indicated for patients categorized as very-high-risk patients according to the National Comprehensive Cancer Network (NCCN) risk classification or whose pathological finding included the component of Gleason grade 5 in our institute, and such cases were performed by TP; therefore, no patient in this study underwent ePLND. The severity of perioperative complications was evaluated according to the Clavien-Dindo system [14], and major complications were defined as those corresponding to Clavien-Dindo ≥3. RARP with EP using HSRS was performed on seven consecutive cases, and patients’ characteristics and their perioperative outcomes were compared with those of 14 cases of RARP with TP using HSRS performed immediately prior by the same three surgeons.

**Figure 1:**
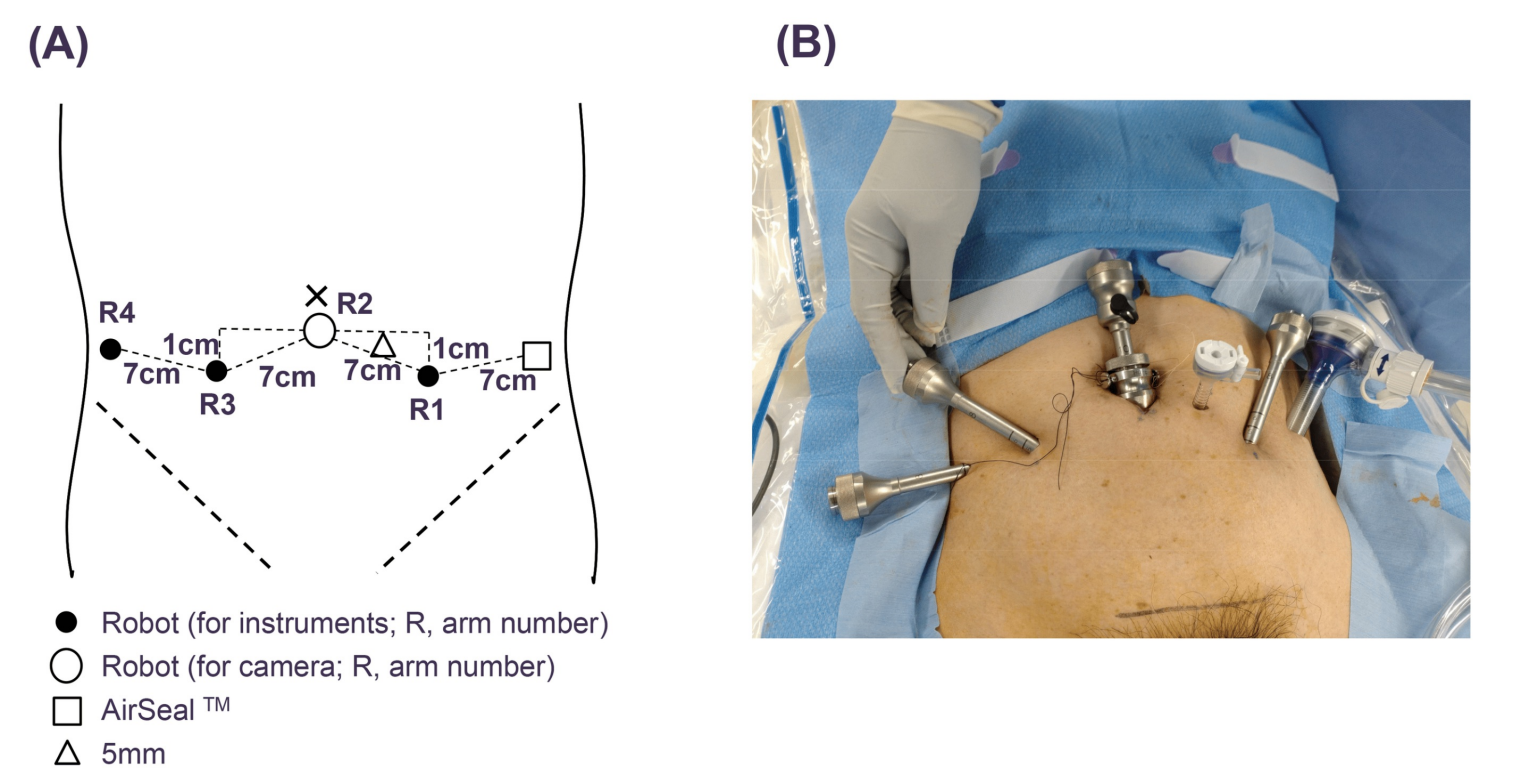
Port positions for the extraperitoneal approach to robot-assisted radical prostatectomy using the hinotori™ surgical robot system. (A) diagram and (B) picture.

A total of three surgeons who had been certified as surgeons using the robotic system for the HSRS participated in this study. The trocar placements are shown in Figure 1.

**Figure 2:**
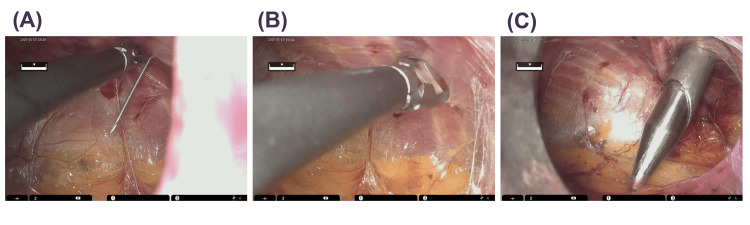
Method of placing trocar in limited space. (A) A 14G needle was inserted to confirm the insertion site, (B) the abdominal wall at the insertion site was dissected using laparoscopic curved dissector forceps inserted through another port, and (C) the trocar was inserted from outside the body using this as a landmark.

Of the three surgeons, two had experience with more than 40 cases of RARP with TP, and the other did not. All surgeons had experience of more than five cases of RARP with TP using HSRS. The port placement in RARP with EP was performed by modifying the procedure for the DVSS mentioned previously [9]. Briefly, with 15 degrees of Trendelenburg position, the camera port was placed by using an open laparotomy procedure, cutting the rectoabdominal fascia and transversalis fascia while avoiding opening the peritoneum. We then created an extraperitoneal space using a balloon dilator, finger assistance, and laparoscopic forceps. Once sufficient extraperitoneal space was secured, the remaining four trocars were placed as shown in Figure 1. First, ports R1 and R2 were placed, and then other ports were placed with the assistance of a laparoscopic curved dissector inserted through these ports, paying attention to avoid damaging the peritoneum (Figure 2).

RARP was performed following standard surgical techniques [15]. Briefly, after cutting the endopelvic fascia, the prostate and the seminal vesicles were dissected in an antegrade manner. In accordance with our therapeutic policy, the nerve-sparing technique was not used in cases with the following findings: Prostate Imaging Reporting and Data System (PI-RADS) [16] score 4-5 lesions identified by preoperative magnetic resonance imaging (MRI), a positive biopsy core with cancer tissue categorized as Gleason grade group 4-5, and palpable induration identified by digital examination. After posterior musculofacial reconstruction, vesicourethral anastomosis and anterior reconstruction were performed with a running barbed suture using 3-0 VLoc.

The drain was removed on postoperative day two, and on postoperative day seven, the urethral catheter was removed after confirming that there was no leakage using cystourethrography.

The differences in the distribution of categorical variables between the two groups were analyzed using a chi-square test, and when >20% of the expected counts were ≤5, they were analyzed using Fisher’s exact test. The differences in continuous variables between the two groups were analyzed using the Mann-Whitney U test. All statistical analyses were conducted using the Statview 5.0 software (Abacus Concepts, Inc., Berkeley, CA, USA), and p-values lower than 0.05 were considered statistically significant.

## Results

In total, seven and 14 patients underwent RARP with EP and with TP using HSRS during the same period, respectively. The preoperative characteristics of the patients are shown in Table 1.

**Table 1:** Preoperative characteristics of patients who underwent extraperitoneal or transperitoneal robot-assisted radical prostatectomy using hinotori TM surgical robot system. EP: extraperitoneal approach; TP: transperitoneal approach; ASA: American Society of Anesthesiologists; BMI: body mass index; HT: hypertension; DM: diabetes mellitus; NCCN: National Comprehensive Cancer Network a: Values are presented as median. b: Comparison between the extraperitoneal and intraperitoneal approach groups.

No.	Age	BMI	ASA score	Initial PSA	Biopsy Gleason score	cT stage	NCCN risk	Prostate volume	Past history	Nerve-sparing surgery	Lymph node dissection
	(years)	(kg/m^2^)		(pg/ml)				(ml)			
EP group											
1	77	18.9	2	5.044	3+4	2a	intermediate	40	HT, DM	unilateral	none
2	59	21.5	2	5.815	4+3	1c	intermediate	23	HT	unilateral	none
3	68	16.3	1	24.3	4+3	2a	high	60	none	none	none
4	77	22.8	1	6.354	3+3	2b	intermediate	50	none	unilateral	none
5	69	21.9	2	6.288	4+3	2a	intermediate	38	appendectomy	unilateral	none
6	66	23.4	2	4.821	3+4	2a	intermediate	48	type-B hepatitis	unilateral	none
7	76	24.9	2	14.837	3+3	2a	intermediate	19	ileus surgery	unilateral	none
Over all^a^	69	21.9	-	6.288	-	-	-	40	-		--
TP group											
1	73	25.7	2	5.13	4+3	2a	intermediate	28	HT, DM	unilateral	none
2	65	23.8	2	5.75	3+4	2b	intermediate	31	DM	unilateral	none
3	66	19.6	2	6.4	3+4	2a	intermediate	19	appendectomy	unilateral	none
4	67	28.4	2	3.8	3+4	2c	high	30	none	unilateral	none
5	80	19.9	2	12.745	4+3	2a	intermediate	37	none	unilateral	none
6	68	23.8	2	6.073	4+4	2a	high	26	none	none	none
7	75	26.9	2	23.4	4+4	2a	high	50	leukemia, type-C hepatitis	none	none
8	79	21.5	2	4.5	3+4	2a	intermediate	25	HT	unilateral	none
9	78	23.9	2	5.8	3+3	2c	high	38	cholecystectomy, ischemic heart disease	none	none
10	67	21.7	2	13.2	3+4	2a	intermediate	41	DM	unilateral	none
11	70	23.9	2	2.2	3+4	2a	intermediate	46	none	unilateral	none
12	75	26.9	2	8.6	3+4	2a	intermediate	32	none	none	none
13	68	23.4	2	7.298	4+3	2a	intermediate	37	HT, DM	unilateral	none
14	59	21.3	1	5.044	4+3	2a	intermediate	23	HT	unilateral	none
Over all^a^	69	23.8		5.937				31.5			
P value^b^	>0.9999	0.1357	0.2474	0.5758			0.6244	0.2631	0.5743	0.6244	

**Figure 3:**
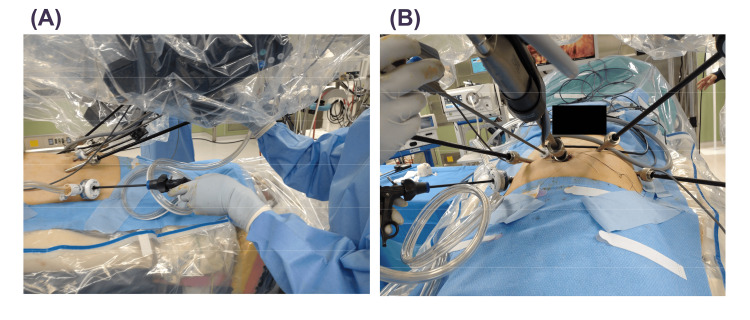
Intraoperative view around the port in extraperitoneal robot-assisted radical prostatectomy using hinotori™ surgical robot system. Pictures of (A) side view and (B) head view.

**Figure 4:**
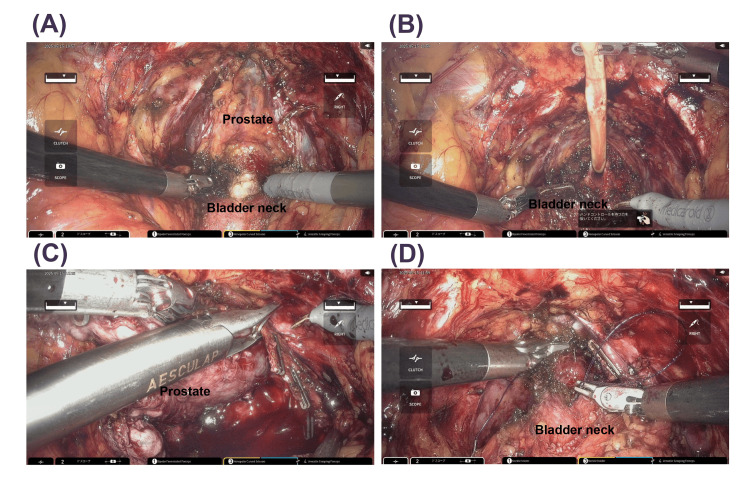
Intraoperative pictures of a patient who underwent extraperitoneal robot-assisted radical prostatectomy using the hinotori TM surgical robot system. (A) Dissection of anterior wall of bladder neck, (B) dissection of posterior wall of bladder neck, (C) dissection of lateral pedicle by intrafascial layer, and (D) vesico-urethral anastomosing suture.

In the EP group, the median age, body mass index, initial prostate-specific antigen (PSA), and prostate volume were 69 years old, 21.9 kg/m², 6.288 ng/mL, and 40 mL, respectively. One and six patients were categorized as high and intermediate risk according to the NCCN risk classification, respectively. All seven patients underwent a nerve-sparing procedure. Since the HSRS is docking-free and the arm is relatively slim, smooth surgical procedures were possible even with narrow port placement in the EP (Figures 3, 4), and there were no major emergency stops due to collision of the arm and forceps.

The major perioperative outcomes for the seven patients are shown in Table 2. The median operative time, time using the robotic system, estimated blood loss, and weight of the resected specimen were 180 minutes, 90 minutes, 100 mL, and 50 g in the EP group, respectively. In case 4, although the measured blood loss, including urine, was high, the postoperative decrease in hemoglobin was minor, and the postoperative course was stable. As a result, no patients required blood transfusion or experienced any major perioperative complications requiring invasive treatment. In all cases of the EP group, the urethral catheter was removed after confirming that there was no leakage from the anastomosis site by cystography on the seventh day after surgery. All cases of the EP group were pathologically diagnosed as T2, and one of all cases (14.3%) showed a positive surgical margin. In the EP group, no significant differences were observed in patient characteristics and perioperative outcomes compared to the TP group (Tables 1, 2).

**Table 2:** Perioperative outcomes of patients who underwent extraperitoneal or transperitoneal robot-assisted radical prostatectomy using hinotoriTM surgical robot system. a: Values are presented as median.

No.	Operative time	Time using robotic system	Estimated blood loss	Weight of resected specimen	Pathological T stage	Pathological Gleason score	Surgical margin status	Indwelling urethral catheter	Complication requiring invasive treatment
	(min)	(min)	(mL)	(ml)				(days)	
EP group									
1	266	172	100	50	2a	3+4	negative	7	none
2	201	128	50	38	2b	4+3	negative	7	none
3	165	96	50	57	2c	4+3	negative	7	none
4	270	175	1270	60	2a	3+4	positive	7	none
5	167	82	200	50	2a	3+4	negative	7	none
6	180	95	200	49	2c	3+4	negative	7	none
7	160	78	10	40	2a	3+4	negative	7	none
Over all^a^	180	96	100	50	-	-	-	7	-
TP group									
1	156	88	200	54	2a	4+3	negative	7	none
2	159	93	250	52	2b	4+3	negative	7	none
3	220	130	200	33	2a	3+4	negative	7	none
4	216	109	100	44	3a	3+4	negative	7	none
5	226	135	25	47	3a	4+3	negative	8	none
6	212	102	200	44	2a	4+3	positive	7	none
7	127	68	0	60	2a	5+4		7	none
8	139	63	20	34	2a	3+4	negative	7	none
9	287	191	700	48	2a	3+4	positive	8	none
10	239	168	50	56	3b	4+3	negative	7	none
11	275	205	100	39	2a	3+4	negative	7	none
12	137	62	0	44	2a	3+4	negative	7	none
13	154	81	10	50	3a	3+4	positive	7	none
14	201	128	10	38	2b	4+3	negative	7	none
Over all^a^	207	106	75	46					
P value^b^	0.6815	0.8520	0.5506	0.3706			>0.9999	0.6015	

## Discussion

In this study, we report the perioperative outcomes for the initial experience of RARP with EP using the HSRS. To the best of our knowledge, this is the first report of RARP with EP using the HSRS.

In RARP with TP, a steep head-down position is required, which raises concerns about circulatory dynamics and adverse effects on glaucoma [12,13], whereas in RARP with EP, this steep head-down position is not required. In addition, EP-RARP is less susceptible to the effects of intra-abdominal adhesions in cases with a history of abdominal surgery. Because of these advantages of EP over TP, previous studies have reported that EP-RARP is equivalent to RARP with TP [10,11,17]. Furthermore, EP has been introduced into RARP using new robots such as Hugo [18], following the DVSS. Modifications of surgical techniques, such as single-port surgery, have been reported [19]. EP requires safe trocar placement and smooth operation in a limited space compared with TP. Since the HSRS was the first domestically produced surgical robot system, its introduction was an extremely significant topic for urologic physicians in Japan. An observational study of 30 cases of RARP using the HSRS showed comparable perioperative outcomes to those of the DVSS [20]. However, there are several points in which the HSRS differs from the DVSS. For example, the robotic arms in the HSRS have eight axes of motion, which is one more than those of the DVSS. In addition, the trocar positioning is calibrated by software without docking an arm with a port. Last, a full high-vision 3D viewer with a 16:9 wide view is mounted in the surgical cockpit. While these characteristics of the HSRS can provide robotic surgeons with technical advantages, these surgeons often feel that there are disparities between the models because many of them are accustomed to surgical procedures using the DVSS. To overcome this, the software of the HSRS has been continually modified and upgraded since its development and introduction. We previously demonstrated that perioperative outcomes were improved by modifying the software of the HSRS [15]. On the basis of the results, we decided to perform RARP with EP using the HSRS, considering that the use of the HSRS enables precise manipulation in the EP space. Since RARP is a widely used technique in TP, we started the procedure not by a single surgeon but by multiple surgeons, even from the initial cases. As shown in Figure 1, the 5-mm port for the assistant was placed between the camera port and the right-hand robot port, so the distance between the ports for the robot on both sides was short, and there were concerns about interference with the robot arm/instruments, but in fact, smooth forceps operation was possible (Figures 3, 4). The fact that the robotic arm is relatively slim and does not easily interfere with the assistant is one of the features of the HSRS and is an advantage that the HSRS offers for the smooth introduction of EP. It is very important to set up the appropriate ports in EP. All procedures by assistants are important, and among them, the ability to perform proper suctioning at all times was considered particularly crucial. In this study, we were able to do this smoothly by modifying the method used in previous papers [9], as shown in Figure 2. Since the perioperative outcomes were equivalent to those of RARP with TP (Tables 1, 2) and there were no serious complications and no conversion to other procedures, including open surgery or TP, RARP with EP seemed to be acceptable. However, one case of heavy bleeding was observed. This was because the head was further lightened, and because the pelvis in this case was very narrow, making it difficult to perform apex dissection, hemostasis, and urinary bladder anastomosis. In EP, the space for the bag containing the excised tissue is relatively small, so vesicourethral anastomosis is performed with the bag secured cephalad by a versatile grasper. Therefore, it is important to pay attention to the proper development of the operative field. Regardless of the variation in cases, it is important to establish a technique that ensures safety and stable perioperative outcomes and to appropriately evaluate preoperative cases.

Despite the promising results, several limitations of this study should be noted. First, this was a small retrospective case series. In addition, the experience of the surgeons using the robotic system inevitably affected the results. Third, we usually performed extended PLND in patients who were at high risk or higher on the basis of the NCCN risk classification, and we did not choose EP for these patients. Finally, postoperative long-term observation should be required to more accurately characterize both oncological and functional outcomes of RARP with EP using the HSRS.

## Conclusions

In conclusion, this is the first study to report the perioperative outcomes of RARP with EP using the HSRS. In this series, a total of seven patients were included, and RARP could be successfully completed as planned in all seven cases without severe complications, resulting in acceptable perioperative outcomes. However, to gain definitive conclusions on several issues assessed in this study, a prospective study including a sufficient number of patients should be carried out in the future.
